# Socioeconomic impacts of COVID-19 pandemic on foodborne illnesses in the United States

**DOI:** 10.29333/ejeph/12585

**Published:** 2022-10-28

**Authors:** Luma Akil, Hafiz Anwar Ahmad

**Affiliations:** 1 Department of Behavioral and Environmental Health, College of Health Science, Jackson State University, Jackson, MS, USA; 2 Department of Biology, College of Science, Engineering and Technology, Jackson State University, Jackson, MS, USA

**Keywords:** foodborne diseases, COVID-19, FoodNet, pandemic, food safety

## Abstract

Foodborne diseases continue to impact human health and the economy. The COVID-19 pandemic has dramatically affected the food system from production to consumption. This project aims to determine the impact of the COVID-19 pandemic on the spread of foodborne diseases and the factors that may have contributed, including environmental, behavioral, political, and socioeconomic. Data for this study were collected from The Foodborne Diseases Active Surveillance Network (FoodNet) for 2015-2020. FoodNet personnel located at state health departments regularly contact the clinical laboratories in Connecticut (CT), Georgia (GA), Maryland (MD), Minnesota (MN), New Mexico (NM), Oregon (OR), Tennessee (TN), and selected counties in California (CA), Colorado (CO), and New York (NY). Data were analyzed using SAS to determine the changes in rates of foodborne pathogens reported in FoodNet before and during the COVID-19 pandemic in the ten reporting states. Results of the study showed a significant decline in the incidences of foodborne diseases ranging between 25% and 60%. A geographical variation was also observed between California and states with the highest decline rate of foodborne illnesses. Policies and restrictions, in addition to environmental and behavioral changes during the COVID-19 pandemic, may have reduced rates of foodborne diseases.

## INTRODUCTION

The World Health Organization (WHO) declared the outbreak of the infectious disease COVID-19 as a pandemic on March 11, 2020 [1]. The virus was identified in Wuhan (China) and has spread worldwide, resulting in more than 581 million cases and over 6.4 million deaths [2]. According to the Centers for Disease Control and Prevention (CDC), the virus that causes COVID-19 is thought to spread mainly from person to person through respiratory droplets produced from coughs, sneezes, or talks of the infected person [3]. The virus impacted populations of different ages and clustered in older folks [4]. Most of the infected countries took strong containment measures to slow down the transmission of the virus. Some of these measures include restrictions on daily living such as home quarantine, social distancing, temporary closing of businesses, schools, and universities, and remote working [5]. While these measures are vital to stop the spreading of COVID-19, they had a significant impact on agriculture and food systems [5].

Even though COVID-19 transmission through food products has been minimal [3], agricultural and food markets faced disruptions from this pandemic. These impediments were due to labor shortages created by restrictions on movements of people and shifts in food demand resulting from closures of restaurants and schools and income losses. Most of these disruptions result from policies adopted to contain the spread of the virus. The pandemic is affecting the food security, availability, access, utilization, and stability of food products [6, 7]. The COVID-19 pandemic has impacted access to food, caused shifts in consumer demand toward cheaper, less nutritious foods, and food price instability, especially among minority and underserved communities [8, 9]. Such deficiencies may lead to food safety and increased disease transmission through food products. Such an increase in foodborne diseases may result from agricultural practice, shortage of employees in restaurants, food preparation, and delivery methods. Human cooking behaviors and hygiene practices may also play a role. In 2020, CDC investigated at least ten multistate foodborne outbreaks caused by *Salmonella, Ecoli, Listeria*, and other pathogens. These outbreaks resulted in thousands of cases of illnesses [10].

In this study, we aim to understand the impact of the COVID-19 pandemic on food safety and the spread of foodborne diseases. We examined the geographical, social, and economic variables that may impact the spread of foodborne diseases during the pandemic.

## METHODS

Data for the study were collected from the Foodborne Diseases Active Surveillance Network, or FoodNet, to identify the most frequently reported diseases and assess the risk factors contributing to foodborne illnesses. FoodNet is a collaborative project of the CDC, the EIP network, the US Department of Agriculture (USDA), and the Food and Dmg Administration (FDA). CEIP’s FoodNet project collaborates with local and state health jurisdictions to implement active surveillance and epidemiologic studies designed to help public health officials better understand foodborne diseases in the United States [11]. The FoodNet conducts surveillance for *Campylobacter, Cyclospora, Listeria, Salmonella, Shiga toxin-producing Escherichia coli (STEC) O157 and non-O157, Shigella, Vibrio*, and *Yersinia* infections diagnosed by laboratory testing of samples from patients. Ten FoodNet sites nationwide serve as a network for responding to new and emerging foodborne diseases of national importance. The FoodNet personnel located at state health departments regularly contact the clinical laboratories in Connecticut (CT), Georgia (GA), Maryland (MD), Minnesota (MN), New Mexico (NM), Oregon (OR), Tennessee (TN), and selected counties in California (CA), Colorado (CO), and New York (NY).

The data from the FoodNet was collected for all reported diseases and states from 2015 through 2020. The years 2015-2019 were used as a pre-pandemic period, and 2020 was an early stage of the pandemic. In addition, data were collected from the United States Census Bureau Business, and Industry Data [12]. Data of the advanced monthly sales for retail and food service for retail trade and food service and food and beverage store sales from 2015-2020 were collected [13]. Further, data from the United States Department of Agriculture (USDA) Food Expenditure Series were collected for the monthly sales of food at home (FAH) and food away from home (FAFH) from 2015-2022 [14]. Husch Blackwell, State-by-State COVID-19 Guidance [15] was used to summarizing the state’s policies and regulations during the pandemic.

### Data Analysis

Data from FoodNet for all reported diseases and states were analyzed using SAS 9.4. Analysis of variance, t-test, and time series analysis was carried out to determine the significant difference in rates of foodborne diseases over time and the change of rates during the pandemic year of 2020, the rates between the states, the seasonal and monthly variation of rates, besides the racial, age and gender differences. Correlational analysis was conducted to determine the association between the economic variable, such as money spent at retail and food service, and the money paid for preparing food at home and away from home.

## RESULTS

**Figure 1.**
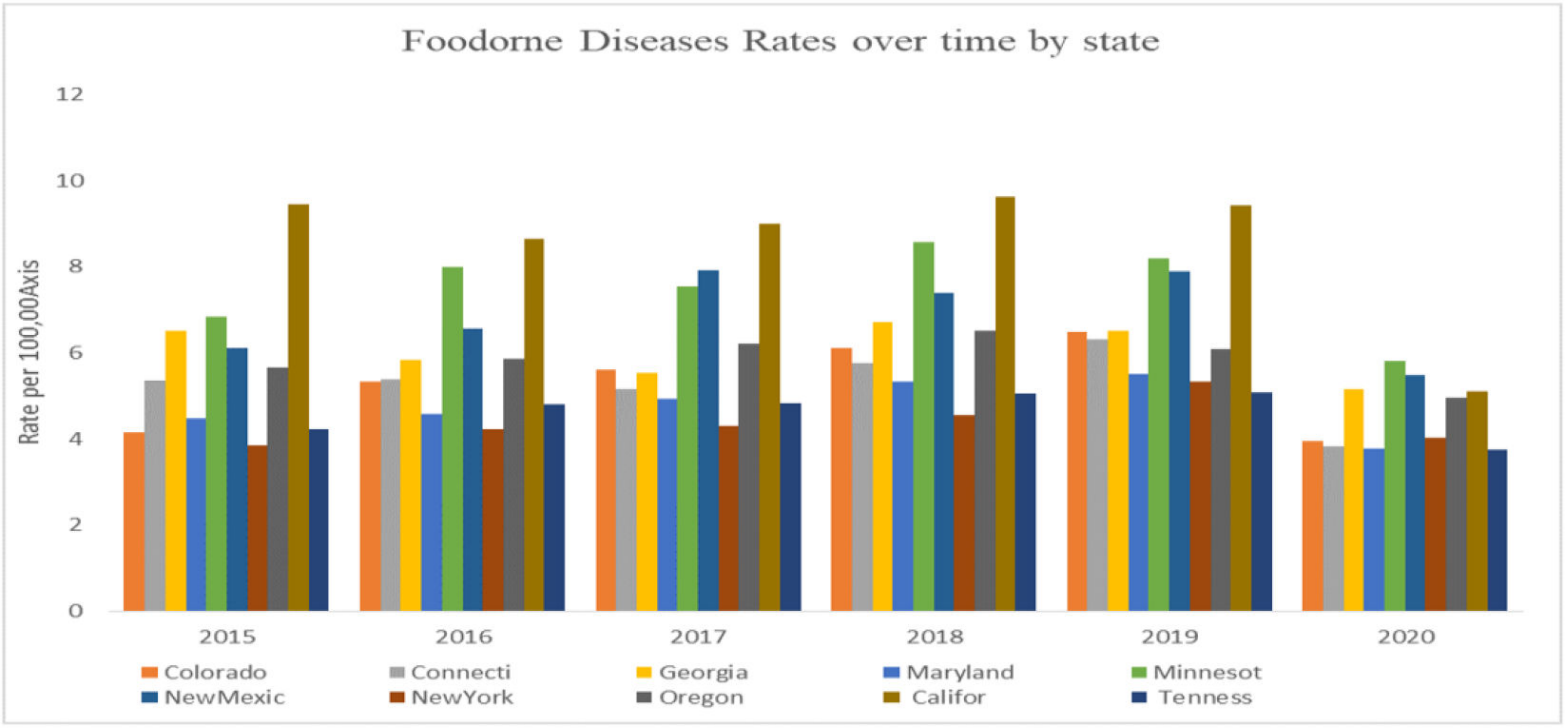
Rates of reported foodborne diseases in FoodNet sates from 2015-2020 (Source: Authors’ own elaborations)

Results of this study showed a significant geographical variation between the states that report to the FoodNet. Overall, CA showed the highest rates of foodborne diseases (average of 8.54/100,000 cases per year) during the study period except in 2020, where MN showed the highest rates of foodborne diseases, as shown in Figure 1. TN and NY had significantly lower rates of foodborne diseases throughout the study period, with an average of 4.62/100,000 and 4.38/100,000 cases per year, respectively (p<0.01).

**Figure 2.**
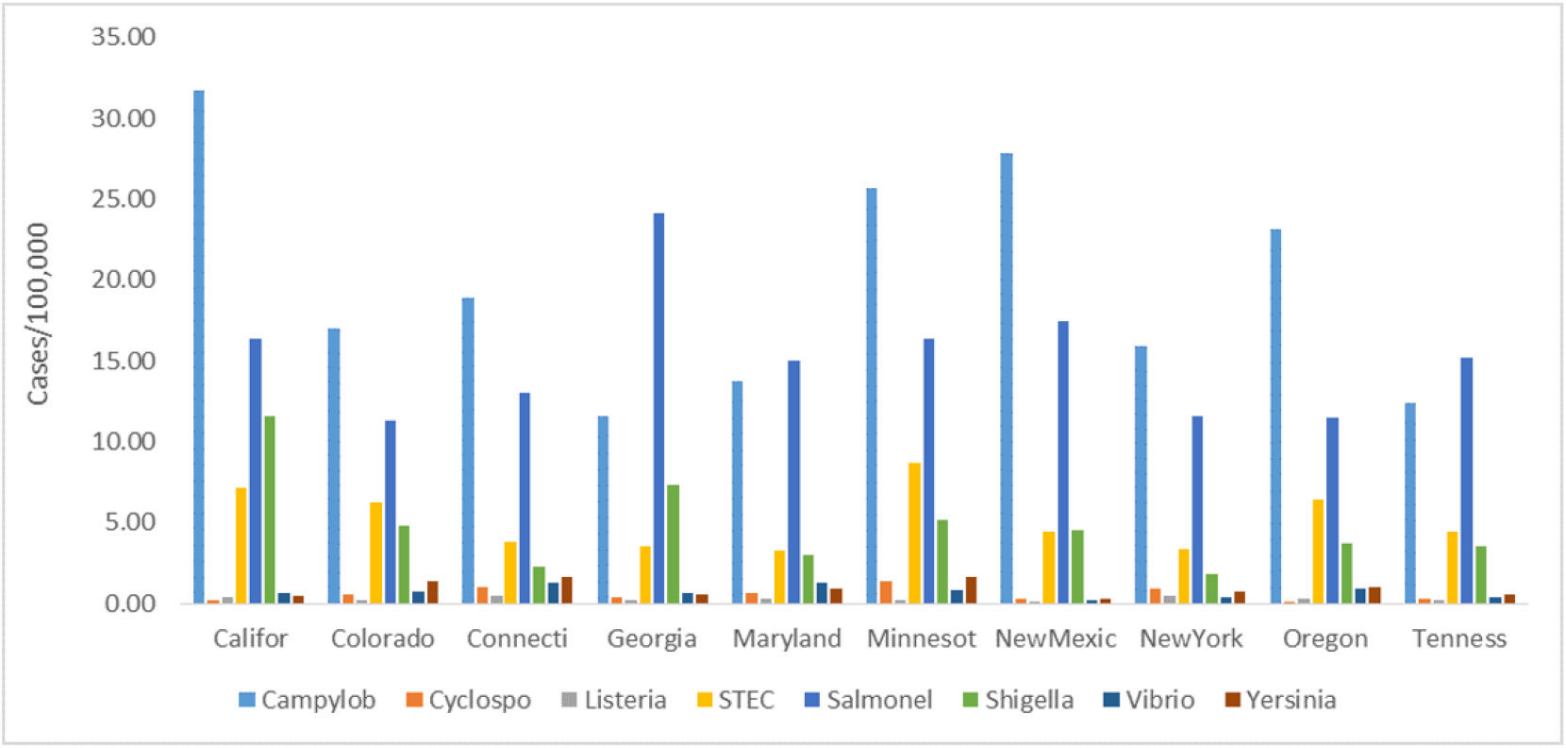
Rates of reported foodborne diseases by state from 2015-2020 (Source: Authors’ own elaborations)

During the study period, *Campylobacter* and *Salmonella* remained the highest reported foodborne diseases with an average of 19.81/100,000 and 15.20/100,000, respectively. Listeria had the lowest reported cases of foodborne illnesses, with an average of 0.27/per 100,000. The highest rates of *Campylobacter* were reported in CA, with an average of 31.7/100,000 cases per year, followed by NM, with an average of 27.85/100,000 cases. The highest reported cases of *Salmonella* were in GA, with an average of 24.18/100,000 cases, followed by NM, with an average of 17.44/100,000 cases per year (Figure 2).

Further, the results showed a significant decline in most foodborne pathogens in 2020 for all ten reporting states (p<0.01). The highest average of foodborne diseases for the states was in 2019 (6.68/100,000 cases), and the lowest was in 2020, with an average of 4.58/100,000 cases.

**Figure 3.**
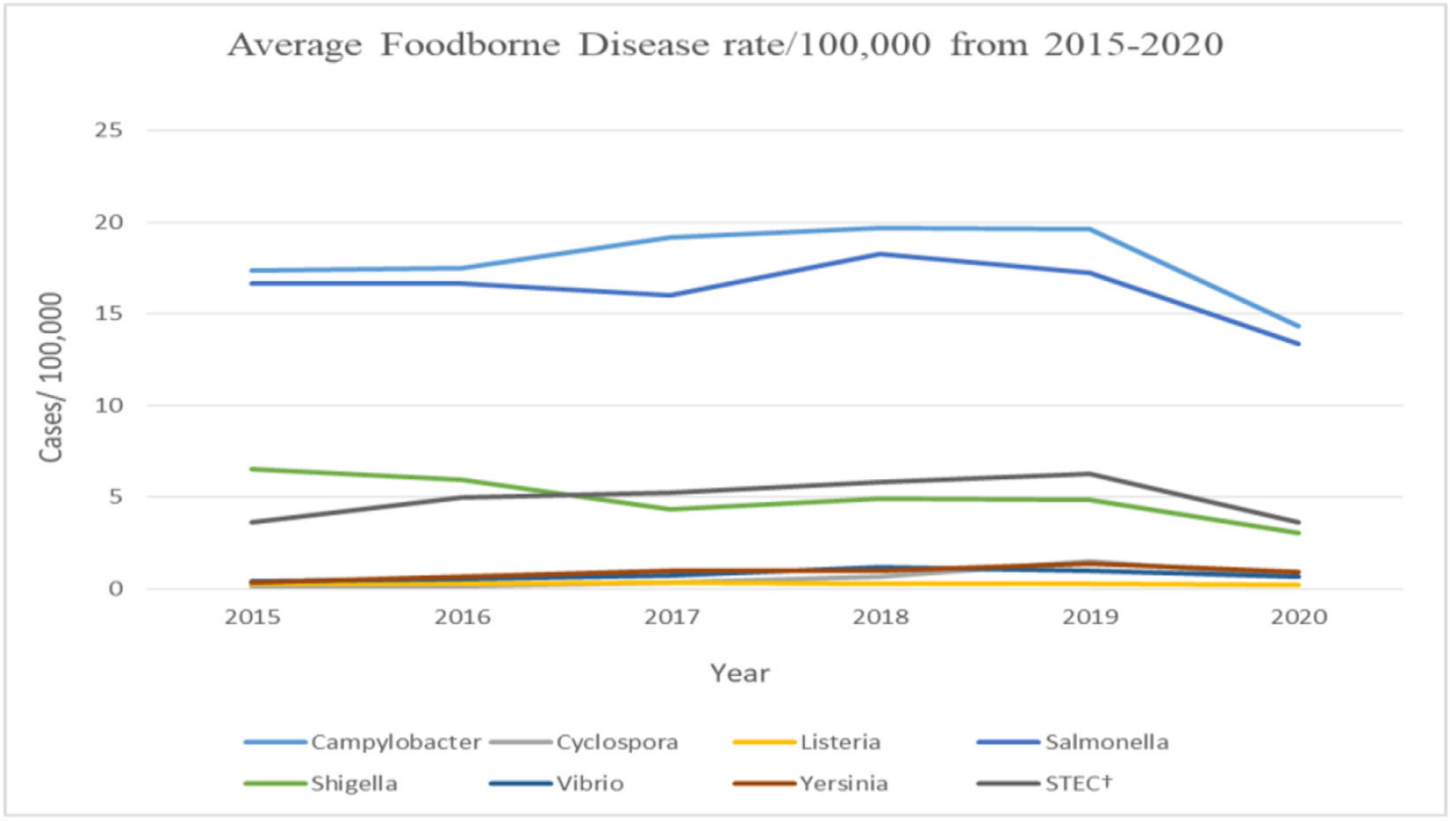
Campylobacter and salmonella were the highest reported foodborne diseases from 2015-2020 (Source: Authors’ own elaborations)

The highest decline in foodborne disease rates was in CA, averaging 61%, while in TN, foodborne diseases only declined by about 25%. A significant decrease in *Campylobacter* and *Salmonella* rates was observed, averaging 35% and 32%, respectively. Further, the highest decline in foodborne rates was in *Shigella* and STEC, with an average of 60% and 55% in the reporting states, respectively (Figure 3).

**Figure 4.**
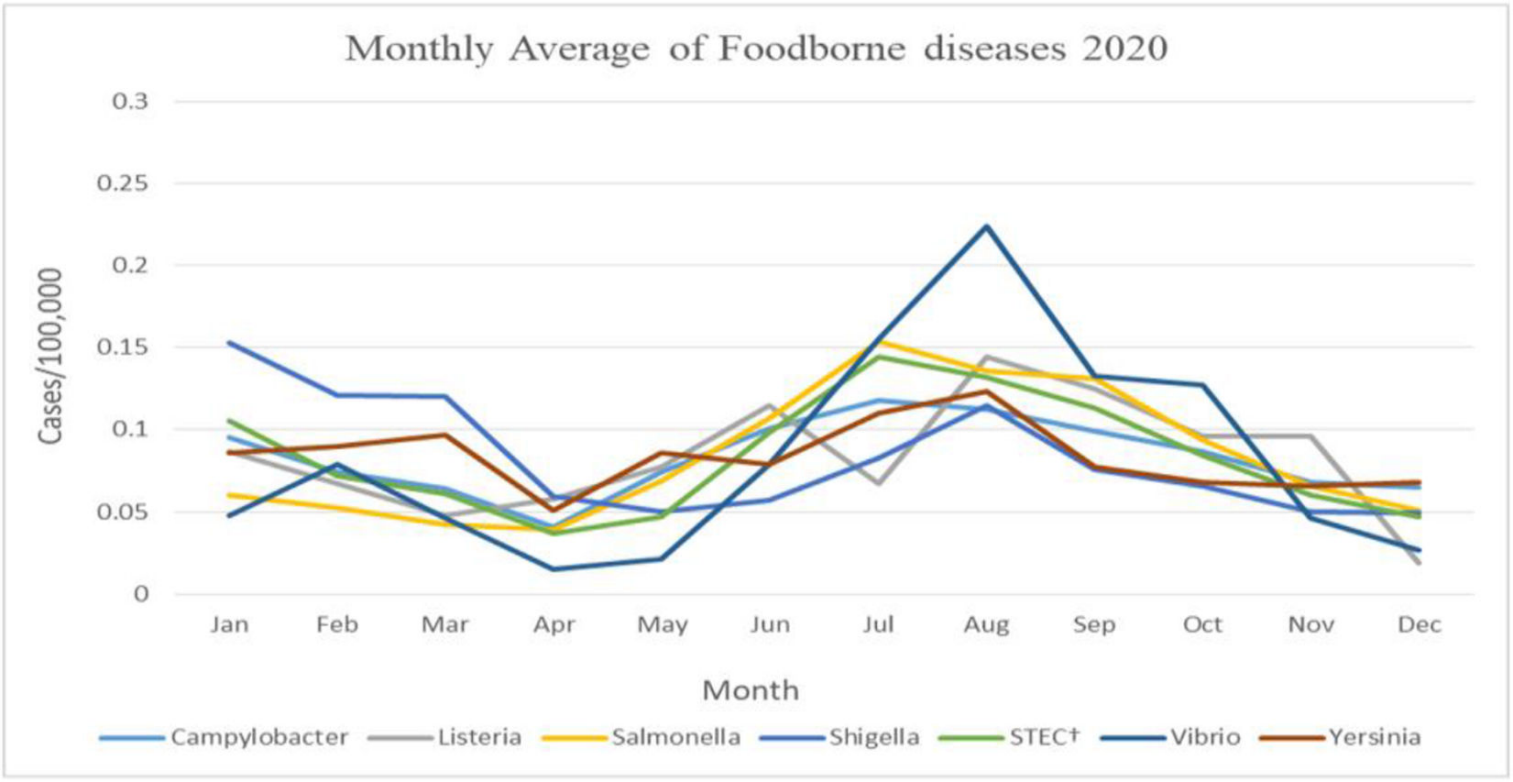
Monthly rates of foodborne diseases in 2020 (Source: Authors’ own elaborations)

In 2020, a seasonal and monthly change was observed in the rates of foodborne diseases with a similar trend to the previous years. The rates of foodborne illnesses were the lowest in March and April of 2020, the rates significantly increased starting May 2020, and a summer peak was observed for some pathogens such as *Salmonella* and *vibrio* (Figure 4).

**Figure 5.**
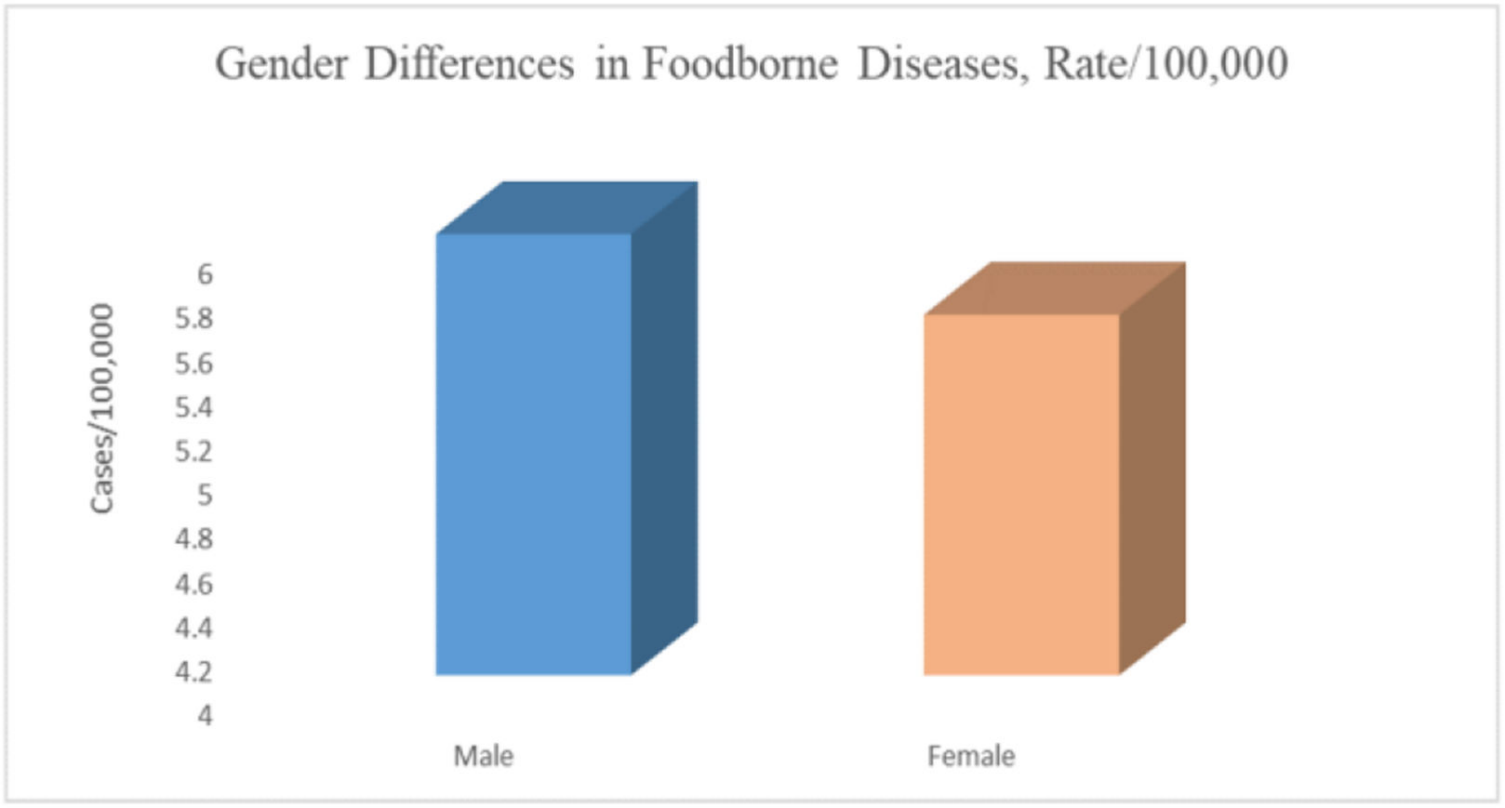
Rates of foodborne diseases by gender (Source Authors’ own elaborations)

Analysis of the demographics of foodborne disease cases showed no significant difference between males and females; however, males had slightly h9igher rates than females, 6.0/100,000 and 5.6/100,000 cases, respectively (Figure 5).

**Figure 6.**
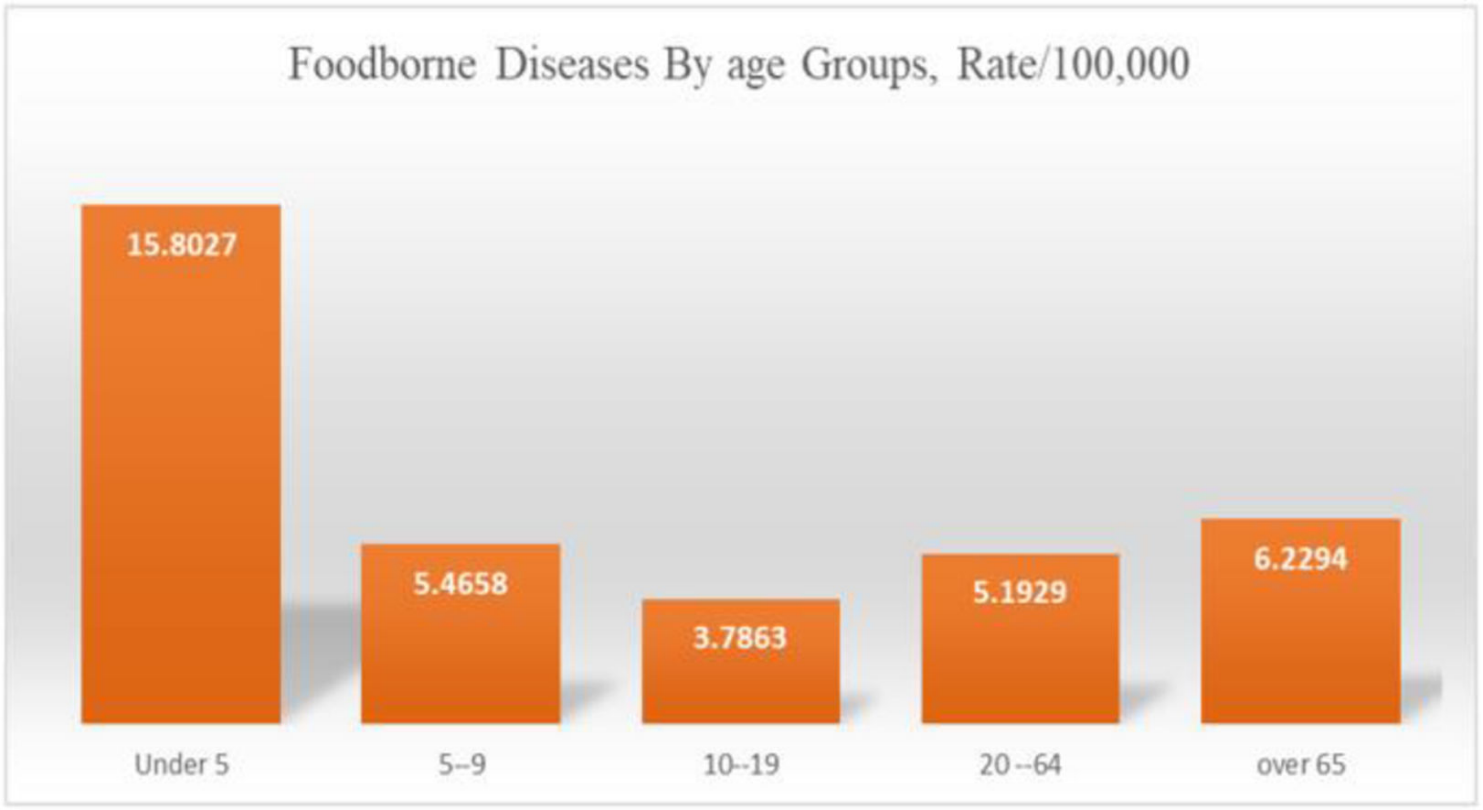
Rates of foodborne disease by age groups (Source Authors’ own elaborations)

In addition, a higher incidence of foodborne diseases was more common among children under five and the elderly above 65 years (Figure 6).

**Figure 7.**
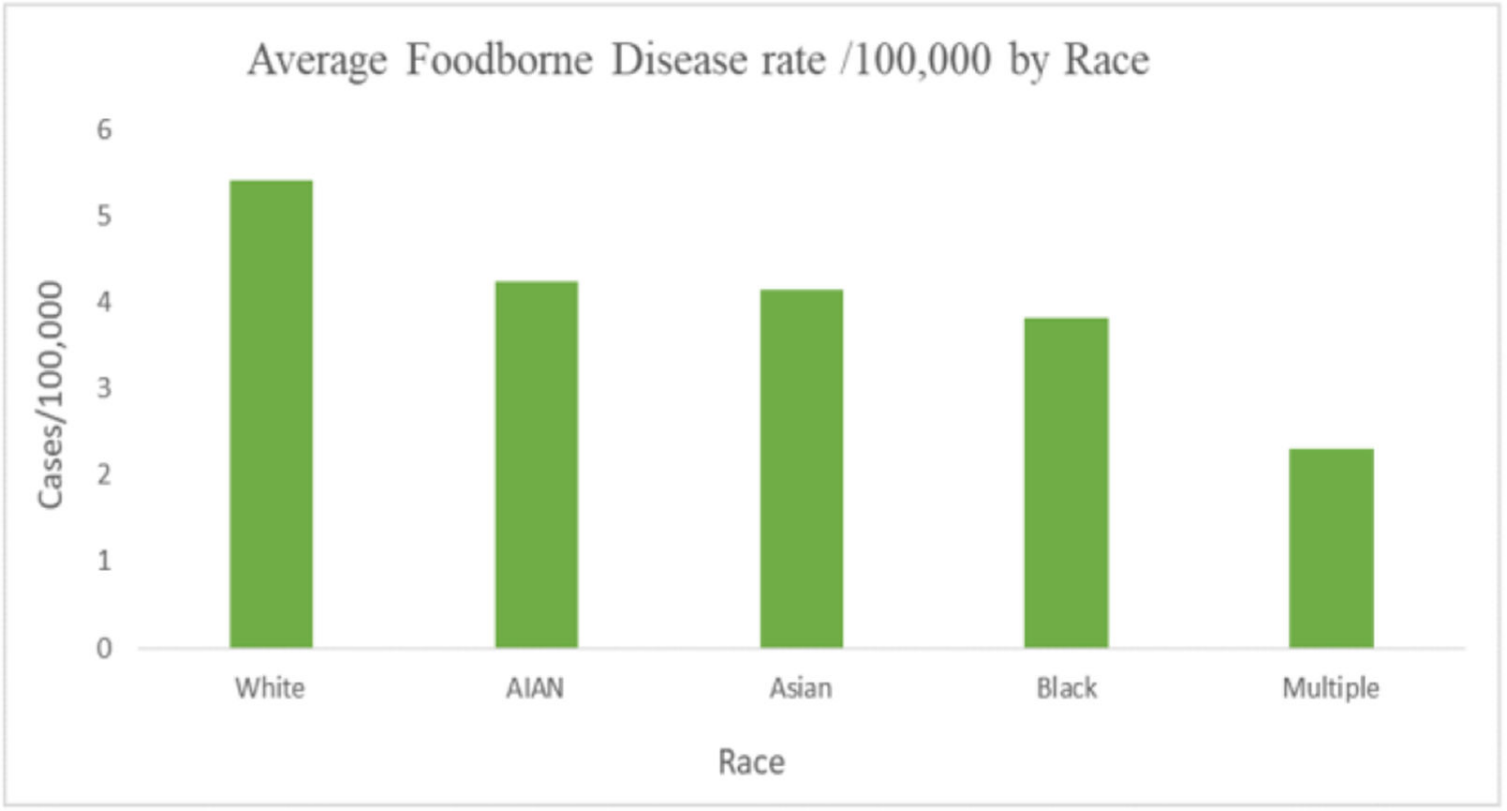
Rates of foodborne diseases by race (Source: Authors’ own elaborations)

White populations showed a higher incidence of primary foodborne diseases than other racial groups, with an average of 5.4/per 100,000 cases. The Black population reported the lowest, with an average of 3.8/100,000 cases (Figure 7).

To understand the implications of each state’s policies and regulations on the spread of foodborne diseases, we examined the state’s policies regarding closures of businesses and stay-at-home orders and regulations, the number of retail and food services, and the dollar amount of spending on eating at home and away from home. A summary of the 10 states’ policies and regulations is shown in Appendix A.

We found that states with more strict laws and delayed opening, such as CA, showed the highest decline in foodborne diseases. In contrast, states such as TN that had early beginnings of businesses and restaurants showed the least drop in foodborne diseases.

**Figure 8.**
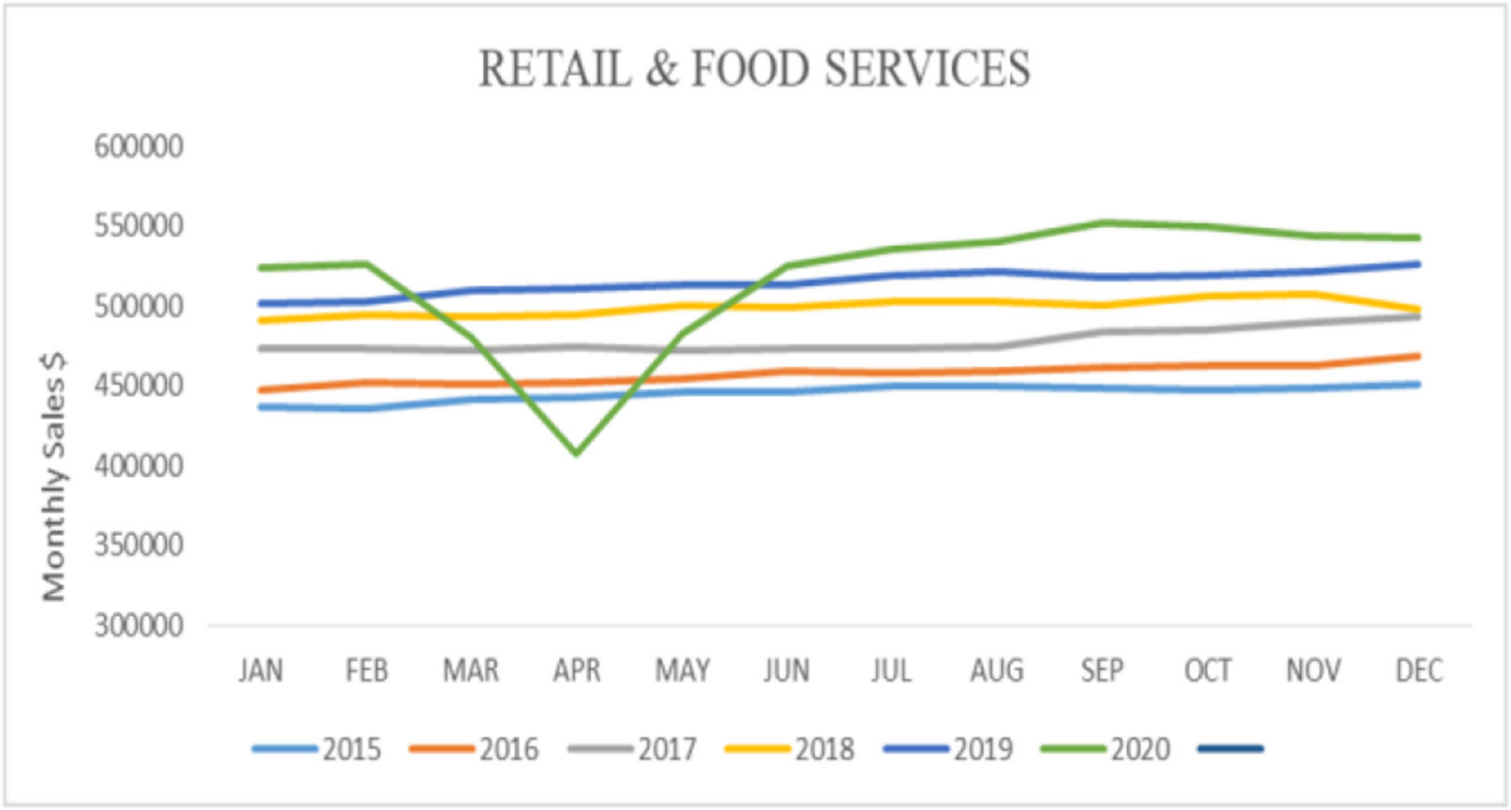
Monthly sales for retail and food services (Source: Authors’ own elaborations)

A significant decline in the number of retails and food services was observed, especially during the early months of the pandemic (Figure 8).

**Figure 9.**
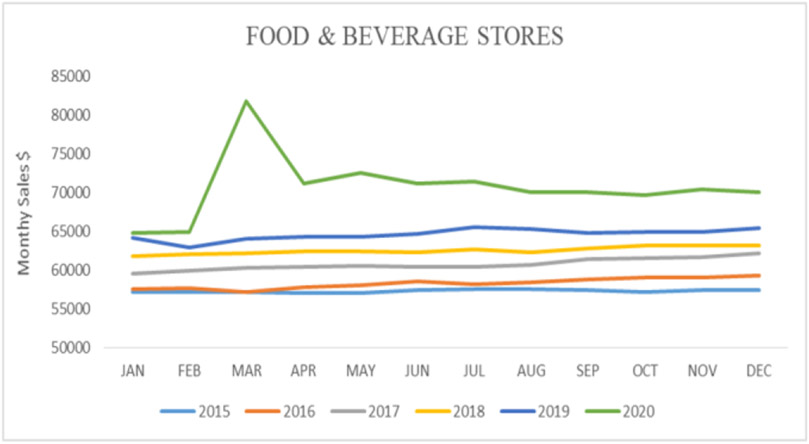
Monthly sales for food and beverage stores (Source: Authors’ own elaborations)

In addition, people spent more money eating at home vs. away from home in 2020, as shown in Figure 9.

Results also showed a moderate correlation between foodborne diseases and the sales for retail and food services (*r*=0.55; *y*=790831*x*+451652). At the same time, there was a low negative correlation between foodborne diseases and the sales from food and beverage stores (*r*=−0.17; *y*=−25608*x*+72798).

**Figure 10.**
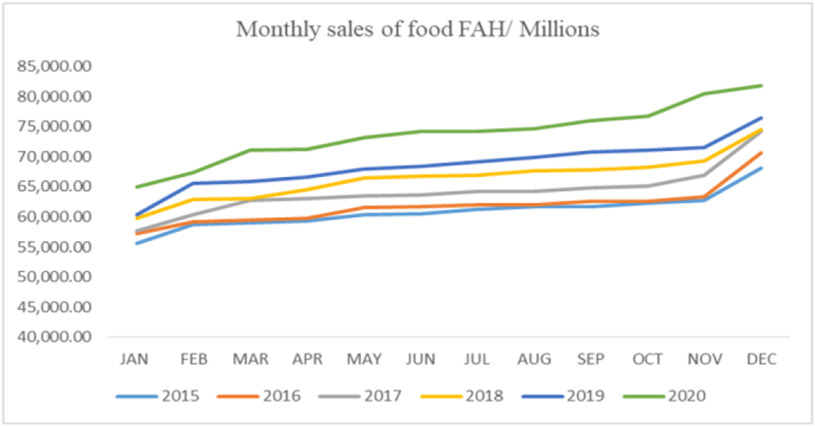
Monthly sales of food at home (Source: Authors’ own elaborations)

In 2020 people spent more money eating at home with an average of $73,823, which was significantly lower than the previous years of $64,414 (Figure 10).

**Figure 11.**
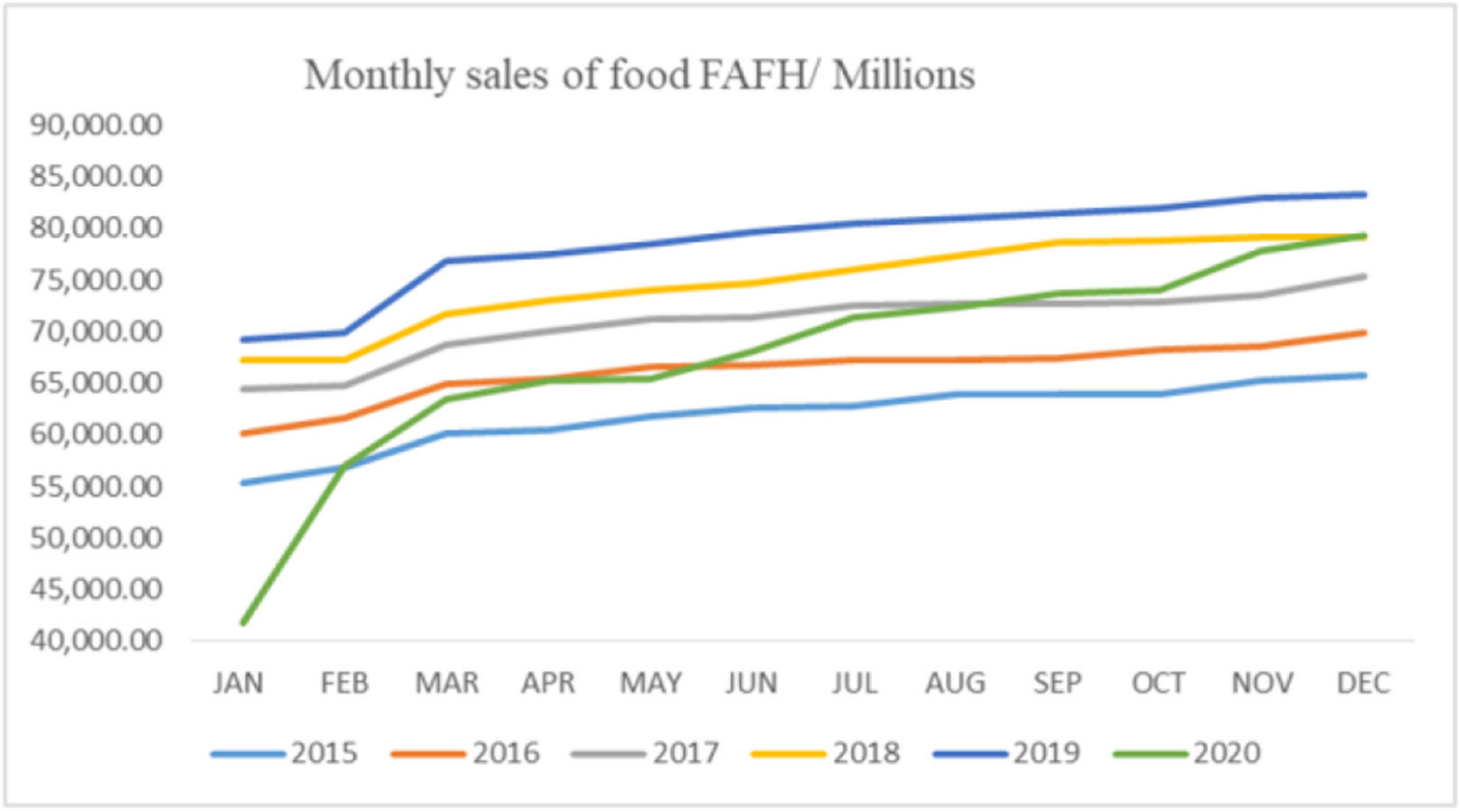
Monthly sales of food away from home (Source Authors’ own elaborations)

However, people spent $67,480 eating away from home in 2020 compared with the average of $70,449 (Figure 11). A weak negative correlation was observed between spending money eating at home and the rates of foodborne diseases in 2020 (*r*=−0.10).

## DISCUSSION

In this study, we aimed to understand the impacts of the COVID-19 pandemic on the spread of foodborne illnesses and its association with environmental, behavioral, social, economic factorsand geographical variations. During the pre-pandemic period (2015-2019), the highest cases of foodborne diseases were observed in CA, a highly populated state, with over 39 million people residing there [16]. CA is also a state with high pollution levels and has suffered from climatic changes such as drought and fires. High pollution levels, seasonal drought, and climate may lead to the spread of foodborne diseases due to changes in farming and agricultural practices. Several foodborne diseases are reported due to contamination of fresh produce and animal products from polluted sources with pathogenic bacteria, viruses, and protozoa [17-19]. These pathogens could be introduced to the foods of animal and non-animal products during primary production, at harvest and slaughter of animals, transportation, food processing, storage, distribution, and preparation, and serving [18].

The highest reported pathogens during our study were *Salmonella* and *Campylobacter*. Both pathogens can infect humans by consuming contaminated or undercooked poultry or meat products [20]. Several ecosystem hazards, including climate change, contaminated water, excess fertilizers-pesticides, poor sanitation, and dissemination of carriers of foodborne pathogens such as insects and rodents, fused with changes in weather conditions, may lead to such diseases [21, 22]. Environmental exposure to climate change is directly linked to changes in the distribution of pathogens resulting in foodborne diseases. For example, *Salmonella* increases as temperature increases [23]. Contamination of oyster beds has been linked to heavy rainfall events, and warming ocean water has led to the expansion of *Vibrio parahaemolyticus* outbreaks due to oyster consumption [24].

Furthermore, in CA, about 61.6 % of the population are White, 12.4% are Black, 18.7 % are Hispanic, and 6% are Asians [16]. In our study, Whites report the majority of foodborne diseases cases. In our previous study, we observed a similar trend in the state of Mississippi [20]. Foodborne diseases such as *Salmonella* incidence increased with higher education and income levels. People with higher income levels may have better access to care, more international travel, consumption of high-risk food items, and eating at restaurants which may lead to high rates of foodborne diseases.

During the 2020 pandemic, a significant decline in the rates of foodborne diseases was observed in most states, with CA having the highest decrease in rates of foodborne diseases. A significant reduction in pollution rates was observed during the early stages of the pandemic [25]. In addition, The COVID-19 pandemic introduced unexpected stresses on food systems. Agriculture was significantly impacted by the pandemic resulting from less demand for biofuels, which in turn led to reduced demand for grains used in biofuels. The acute decline in food demand by restaurants and hotels impacted farmers’ sales of food products, especially meat, dairy, and specialty crops, resulting in decreased commodity prices [26]. These reductions in the production of food products have led to lower contamination, especially at the primary production level.

In addition to the environmental and climatic factors, foodborne disease incidences and outbreaks were mainly linked to restaurant settings. Studies have shown that more than half of all foodborne disease outbreaks reported to the CDC are associated with eating in restaurants [27]. *Norovirus* and *Salmonella* are the two most common pathogens accounting for nearly 75% of outbreaks reported in the United States. They are associated with restaurant outbreaks, mainly through transmission by food workers [27, 28]. Practices such as pooling eggs, handling and storing foods at a temperature that helps low-dose pathogens amplify, undercooking meat products, and cross contamination of cooked food will lead to such outbreaks [21, 29]. According to The National Restaurant Association, 47% of every dollar spent on food was in a restaurant in 2016, and the average American ate out approximately five times per week in 2015 [30]. The percentage of spending on food eaten away from home has increased during recent decades. However, our study showed a significant decline in eating at restaurants and away from home during the early months of the pandemic due to strict preventive measures. The closure of restaurants and food service providers in schools, hotels, and catering businesses has resulted in more eating at home than away from home. Such shift resulted in lower rates of foodborne illnesses, especially the pathogens associated with restaurant settings. Food loss and food waste were major issues during the COVID-19 pandemic [31]. Food loss was a significant risk from production to consumers or wasted by retailers or families resulting from panic and policy adaptations [32].

Restrictions and policies implemented in states such as CA also contributed to the significant reduction in foodborne illnesses. In contrast, we found that, in states such as TN, with earlier openings and fewer restrictions, foodborne diseases showed the lowest decline rates. Further, a shift in demand for food items was observed during the pandemic. The market had shifted away from higher-value items to staple and ready-to-eat foods that can be stored. A significant increase in spending on such food items was observed, especially during the early stages of the pandemic. A study has shown that the decrease in shopping frequency during the COVID-19 pandemic was significantly related to an increase in frozen food and canned food consumption in Germany and Denmark, suggesting some people partly substituted fresh food with frozen food canned food [33]. Reduction in fresh fruits and vegetables, meat, and dairy purchase has also contributed to the decline of foodborne diseases

Furthermore, international travel restrictions have decreased infections associated with such activities [34]. These policies and regulations and changes in hygiene behaviors, such as increased handwashing, likely reduced exposure to foodborne pathogens [35]. Studies have shown that washing hands before preparing food increased by at least 20% during the COVID-19 pandemic [36].

As an overwhelmed healthcare system during the pandemic, factors such as changes in healthcare delivery, health care-seeking behaviors, and laboratory testing practices, might have decreased the detection of enteric infections [34]. Studies have shown a nearly 60% decline in the number of visits to ambulatory practices by early April of 2020, with a decrease in in-person visits and an increase in telehealth visits [37]. In addition, it was reported that the pandemic had affected a wide range of services, including essential services for infectious diseases, non-communicable diseases, mental health, reproductive, maternal, newborn, child, and adolescent health, and nutrition services [38]. These healthcare-related factors may have resulted in declining reported diseases such as foodborne diseases [39].

In conclusion, our findings showed a significant decline in the rates of foodborne diseases during the 2020 and early months of the COVID-19 pandemic. Several factors may have contributed to such a decline in incidences of foodborne diseases. These factors may be environmental, behavioral, political, economic, or social. Preventive measures taken during the pandemic may have also contributed to the reduction of rates of foodborne diseases.

## Data Availability

Data supporting the findings and conclusions are available upon request from the corresponding author.

## APPENDIX A

**Table A1.** States policies during the COVID-19 pandemic March through December 2020

State D/R Policy		
CA	D	3-4-2020 Government issues proclamation of a state of emergency
		3-15-2020 Governor Newsom called for all bars, wineries, nightclubs, and brewpubs to close
		3-19-2020 stay at home order issued
		5-19-2020 the stay-at-home order is managed county by county
		6-1-2020 restaurants and retail stores may open
		6-10-2020 most places are allowed to open with restrictions
		7-1-2020 indoors places are ordered to close if they are on the states watch list for over 3 days
		7-20-2020 schools allowed to open in person
		12-7-2020 Increase in restrictions
CO	D	3-12-2020 restricting the visitation of non-essential individuals
		3-22-2020 executive order directing all of Colorado’s non-critical employers to reduce their in-person workforce by 50 percent.
		3-26-2020 issued stay-at-home orders for the entire state of Colorado
		4-27-2020 implements measures allowing many residents to return to work while maintaining a sustainable level of social distancing
		5-10-2020 entering of the safer-at-home phase
		5-26-2020 safer at home phase and state of emergency extended
		5-28-2020 reopening of restaurants
		6-24-2020 reopening of more services
		7-1-2020 bar and nightclubs ordered to close due to surge in cases
		9-8-2020 protect our neighbors phase allowing schools to open with heavy restrictions
CT	D	3-23-2020 all non-essential functions should suspend all in person interaction
		5-5-2020 all schools remain shut down for the rest of the 2019-2020 school year
		5-20-2020 many places reopening
		6-22-2020 phase 2 or the reopening of most businesses
		7-1-2020 said that phase 3 will begin in mid-July which would allow the reopening of bars
		11-10-2020 returning to phase 2
GA	R	4-8-2020 statewide shelter-in-place order
		4-21-2020 business was set to reopen later in the week
		4-27-2020 most businesses allowed to reopen with restrictions for COVID-19
		5-1-2020 all people 65+ years in age ordered to shelter in place
		5-11-2020 reopening of most businesses including gyms and hair salons.
		5-29-2020 state of emergency, which restricted businesses further
		9-1-2020 state of emergency, which lessened restrictions
MD	D	3-5-2020 declares state of emergency
		3-12-2020 schools closed
		3-16-2020 all bars and non-essential business closed
		3-30-2020 stay at home order
		5-6-2020 ease on the stay-at-home order
		5-14-2020 safer at home order
		5-15-2020 starting of stage 1
		6-3-2020 phase 2 which will begin to open many non-essential businesses
		6-11-2020 announcement that states soon restaurants and gyms will open indoors
		8-3-2020 renewal of state of emergency
		9-1-2020 phase 3
MN	D	3-16-2020 closing down of bars and other non-essential businesses
		3-26-2020 stay at home order
		4-24-2020 extension on distance learning
		5-31-2020 bars and such allowed to open
NM	D	3-25-2020 complete halt on non-essential businesses
		4-23-2020 stay at home order
		5-28-2020 restaurants allowed to open but only outdoors
		6-15-2020 allowing of indoor operations
		6-27-2020 reopening of schools
		8-10-2020 restaurants back to outdoors only
		11-16-2020 stay at home orders
NY	D	3-22-2020 closing of non-essential businesses
		5-5-2020 starting of reopening
		6-2-2020 opening of barber shops
		6-11-2020 starting of phase 3
		6-24-2020 starting of phase 4
		9-10-2020 indoor dining allowed
		11-16-2020 new restrictions for gyms and other indoor areas
		12-8-2020 many liquor stores operating illegally
OR	D	3-8-2020 state of emergency
		3-12-2020 closing of schools
		3-23-2020 stay at home order
		5-8-2020 phase 1
		6-5-2020 phase 2
		6-15-2020 pause on the phase 2 reopening process
		7-29-2020 schools able to open in person for the next school year
		8-17-2020 counties with high cases moved back to phase 1
		11-13-2020 2 week freeze to smiddle the spread
TN	R	3-22-2020 closing of all restaurants
		3-25-2020 safer at home order
		3-31-2020 closing down of businesses
		4-3-2020 stay at home order
		5-6-2020 barber shops and such permitted to open
		5-7-2020 starting of phase 1 for some counties
		5-19-2020 starting of phase 2 for some counties
		6-18-2020 starting of phase 3 for some counties

Note. D: Democratic State; R: Republican State; & Policies & issued regulations during the early phase of the pandemic in the 10 sites of FoodNet
